# JBRA Assisted Reproduction - The scientific journal and the impact factor: metric and non-metric challenges for the editorial board

**DOI:** 10.5935/1518-0557.20230072

**Published:** 2024

**Authors:** Maria do Carmo Borges de Souza, Paulo Franco Taitson, João Batista Alcantara Oliveira

**Affiliations:** 1 Editors JBRA Assisted Reproduction; 2 Scientific Director - Fertipraxis Centro de Reproducao Humana - Rio de Janeiro (RJ), Brazil; 3 Associated Professor - Pontifical Catholic University of Minas Gerais (MG), Brazil; 4 Center for Human Reproduction Prof. Franco Jr - Ribeirão Preto (SP), Brazil

Our journal has completed 27 years of uninterrupted publication, and over the years it represents not only the Brazilian Association of Assisted Reproduction (SBRA) but it also encompassed the Brazilian Association of Embryologists in Reproductive Medicine (PRONUCLEO) and the Latin American Network of Assisted Reproduction (REDLARA). We have evolved into an online, open journal, with a multinational editorial board, constantly striving for quality scientific reporting, without borders or ideologies.

We, the Editors, are flattered that this year we have received our first impact factor (1.5); but we fully understand that the work of the editorial board is continuous and that in order to continue being recognized and read, to still grow in the universe of reproductive medicine, it is necessary to dare and at the same time think critically. Obtaining an impact factor is certainly a great feat; it means we have developed positive actions that have been recognized by the community, with a consequent greater Latin American presence in the scientific environment. The journal is today the first Latin American publication in the field of Obstetrics and Gynecology according to the 2022 Scimago Rank.

However, we also know that just metrics are not enough to grant quality or acceptance, for that matter. A journal must keep a transparent and regular peer review system, with evaluations within defined deadlines and friendly access to the authors. While it must attract studies and publications by authors or groups of researchers, many of them with academic background, it cannot ignore the usefulness and connection with the readers that look for the best guidance and to share experiences. Piccoli (2022) states that clinical and observational studies are rarely unbiased, and retrospective analyses are frequently heavily biased. Meanwhile, they may convey new ideas to be further developed within a more elegant design.

Impact factors are based on the number of highly cited papers. Indeed, their contribution is paramount, but back in 1997 Seglen suggested that 50% of the most cited papers in a journal were cited ten times as often as 50% of the least cited papers, representing an evaluation bias. The system should be fair to all. That is, on the one hand we have the scientist who is looking for a solid and recognized journal to show his study and results, and on the other hand, the possibility of communicating guidelines to help guide associations, as well as presentation abstracts or posters in specialized conventions, and a space for opinions or questions. Is it mandatory to exclude special clinical reports, which may be of general interest, because they do not have an impact?

Within our challenges, we clearly see possibilities for growth, paying more attention to requirements of metrics, without losing this channel of open contact with our readership of specialist professionals. We are working on it and it is up to you, member of our scientific community, to contribute with the papers that will appear in the journal.
